# Influence of *Curcuma longa* extract on cognitive function in mild cognitive impairment due to Alzheimer’s disease: a single-arm, open-label, observational study

**DOI:** 10.3389/fnut.2026.1951923

**Published:** 2026-10-01

**Authors:** Yuta Manabe

**Affiliations:** 1 Division of Dementia and Geriatric Medicine, Department of Advanced Clinical Medicine, Kanagawa Dental University School of Dentistry, Yokosuka, Japan; 2 Dementia Diagnosis Center, Department of Internal Medicine, Yokohama Shintoshi Neurosurgical Hospital, Yokohama-shi, Kanagawa, Japan

**Keywords:** Alzheimer’s disease (AD), bisacurone, cognitive function, *Curcuma longa* (turmeric), mild cognitive impairment (MCI), turmeronol, working memory

## Abstract

**Objectives:**

*Curcuma longa*, commonly known as turmeric, is a dietary spice with potential biological effects. This study evaluated the association between intake of *C. longa* extracts (CLE) and cognitive function in individuals with mild cognitive impairment (MCI) due to Alzheimer’s disease (AD).

**Methods:**

This was a 4-month, single-arm, open-label, observational study of participants with MCI due to AD who were awaiting anti-amyloid therapy. Participants (*n* = 13) consumed the test food containing CLE, which includes the compounds bisacurone and turmeronols. Cognitive function was assessed at baseline and after 4 months using various cognitive assessments.

**Results:**

After excluding participants with insufficient intake of CLE capsules or invalid data, 10 participants were included in the analysis set, consisting of 2 men and 8 women, with a mean ± standard deviation age of 74.8 ± 6.1 years. CLE intake was associated with significantly higher memory scores (Composite B) and significantly lower estimated brain age at 4 months compared with baseline, as measured by a digital cognitive assessment tool (Nou-Know). In the Japanese version of the Montreal Cognitive Assessment, total scores did not differ significantly after CLE intake, but subscale scores for the attention domain and digit span test increased significantly from baseline.

**Discussion:**

This observational study provides preliminary evidence of a potential association between CLE intake and changes in frontal lobe-mediated functions, particularly working memory, in individuals with MCI due to AD, and further investigation is warranted.

**Clinical trial registration:**

https://center6.umin.ac.jp/cgi-open-bin/icdr_e/ctr_view.cgi?recptno=R000058932, identifier UMIN-CTR, UMIN000052043.

## 1 Introduction

Alzheimer’s disease (AD) is the most common cause of dementia. The amyloid cascade hypothesis posits that accumulation of amyloid-β (Aβ) plaques in the brain is a primary event in the development of AD. Within this cascade, Aβ undergoes sequential aggregation, forming oligomers, protofibrils, and fibrils that eventually deposit as senile plaques. In parallel, kinase-mediated hyperphosphorylation of tau proteins promotes the formation of intracellular neurofibrillary tangles, contributing to neuronal degeneration and death. Findings from basic research investigating these mechanisms have led to the development of disease-modifying therapies, including anti-Aβ antibody therapy (AAT) and anti-tau antibody therapy.

Evidence from basic research suggests that *Curcuma longa*, a spice known as turmeric commonly used in curry, may reduce the risk of AD through both direct and indirect effects on Aβ (1–3). Curcumin, a lipophilic polyphenol and major component of *C. longa*, has various physiological effects (4). On the other hand, curcumin-free *C. longa* water extracts have been reported to exhibit diverse biological activities (5). Other constituents of *C. longa* include the sesquiterpenoids such as turmeronols A and B and bisacurone. In the previous preclinical study, turmeronols inhibited the production of inflammatory mediators in macrophages and microglial cells and bisacurone inhibited monocyte adhesion to endothelial cells (6, 7). *C. longa* water extracts, which contain turmeronol A and B, attenuated microglial activation and cognitive impairment in mouse models of AD (8).

An observational clinical study examining the association between cognitive function and curry consumption reported significantly higher Mini-Mental State Examination scores among individuals who frequently consumed curry than those who rarely or never did (9). Although clinical findings regarding the effects of *C. longa* supplementation on cognitive function have been inconsistent, some clinical studies have suggested beneficial effects of *C. longa* on specific cognitive domains, including working memory (10). In addition, recent clinical evidence suggests that *C. longa* extract containing turmeronols A and B and bisacurone may have beneficial effects on cognitive functions associated with orientation and attention in healthy participants with subtle cognitive weaknesses (11). Thus, regular consumption of *C. longa*-containing foods could represent an accessible and cost-effective approach to supporting cognitive function and potentially reducing the risk of AD.

Based on these previous findings, the present observational study was designed to evaluate cognitive function in individuals with mild cognitive impairment (MCI) due to biologically confirmed AD who consumed a test food containing *Curcuma longa* extracts (CLE), which contained turmeronols A and B and bisacurone, while awaiting AAT.

## 2 Materials and methods

### 2.1 Study design

This was a 4-month, single-arm, open-label, observational study conducted from September 2023 to June 2025 at the Dementia Diagnosis Center, Department of Internal Medicine, Yokohama Shintoshi Neurosurgical Hospital in Kanagawa, Japan. The study was registered with the University Hospital Medical Information Network Clinical Trials Registry (UMIN-CTR; registration number UMIN000052043; August 29, 2023). It was conducted in accordance with the principles of the Declaration of Helsinki (2013), the Ethical Guidelines for Medical and Health Research Involving Human Subjects, and the Japanese Clinical Trials Act, following approval from the Ethics Committee of Yokohama Shintoshi Neurosurgical Hospital (approval ID: R5-21; July 4, 2023).

### 2.2 Participants

Eligible participants were diagnosed with MCI [Clinical Dementia Rating Global Score (CDR-GB) of 0.5] due to biologically confirmed AD (diagnosed by amyloid positron emission tomography or cerebrospinal fluid testing) who consumed a CLE-containing test food while awaiting AAT. The objectives and significance of this study were explained to potential participants both verbally and in writing. Informed consent was obtained from all participants prior to enrollment.

### 2.3 Inclusion criteria

Inclusion criteria were as follows:

1. Diagnosis of MCI due to biologically confirmed AD at the study site.
2. CDR-GB score of 0.5, meeting the diagnostic criteria for MCI.
3. Willingness to consume CLE while awaiting AAT.
4. Aged ≥45 and <90 years, with no current or previous malignancy, serious hepatic disease, or other serious internal organ disease.
5. Not pregnant.
6. Provision of written and oral informed consent.
7. Ability to take two capsules of the CLE-containing test food daily, with support from a family member to maintain a diary documenting lifestyle factors and CLE intake.

### 2.4 Exclusion criteria

Exclusion criteria were as follows:

1. CDR-GB score of ≥1.0, meeting the diagnostic criteria for dementia.
2. MCI due to a condition other than AD.
3. Aged <45 and ≥90 years.
4. Current or previous malignancy, serious hepatic disease, or other serious internal organ disease.
5. Regular use of medications, quasi-drugs, or supplements (e.g., docosahexaenoic acid, eicosapentaenoic acid, *Ginkgo biloba* extract, linseed oil, tocotrienols/tocopherols, astaxanthin, gamma-aminobutyric acid, phosphatidylserine, and plasmalogen) that may confound study outcomes.
6. Markedly irregular eating habits.
7. Irregular lifestyle (e.g., shift workers or night-shift workers).
8. Severe visual or auditory impairment.
9. Excessive alcohol intake or alcoholism.
10. Soybean allergy.
11. Planned participation in another clinical study during the study period.
12. Lack of a family member to assist with diary completion.
13. Smoking.
14. Any other condition deemed unsuitable by the investigator.

### 2.5 Test food

In the present study, we used *C. longa,* which was confirmed by an intron length polymorphism marker method targeting the genes encoding diketide-CoA synthase and curcumin synthase, as previously described by Kita et al. (12). The test food was a CLE-containing food provided by House Wellness Foods Corporation (Hyogo, Japan). The capsule composition is shown in Table 1. The daily dose consisted of two capsules with a total weight of 0.96 g, containing turmeronol A (100 μg), turmeronol B (100 μg), and bisacurone (400 μg). Other constituents, including curcuminoids or other sesquiterpenoids, were not quantified. The test food product was standardized to bisacurone and turmeronols, with quality control performed for each production batch to ensure consistency in its composition. The capsule base was composed of gelatin, glycerin, a soybean-derived emulsifier, and beeswax. The capsules contained a mixture of hot water extract and supercritical carbon dioxide extract of *C. longa* (CLE, Turmeric Extract Mixture), prepared as previously described (7, 13, 14). In brief, for the hot water extract, *C. longa* rhizomes were extracted at 98 °C for 1 h; the supernatant was then concentrated, mixed with dextrin, and spray-dried. For the supercritical carbon dioxide extract, rhizomes were extracted at 65 °C and 150 bar for 6 h; the supernatant was then concentrated, mixed with vegetable oil, and filtered through a 1-μm membrane. These two *C. longa* extracts were mixed at a ratio of 8:1 (the hot water extracts to the supercritical carbon dioxide extracts) and the mixture was used as CLE. Safety was confirmed in a previous clinical study in which participants consumed excess doses of CLE (10 capsules per day, i.e., a 5-fold higher dose than in this study), with no adverse effects observed in physical examinations, hematology, biochemistry, or urinalysis (13).

**Table 1:** Composition of the test capsules.

Component	CLE (0.96 g/2 capsules)
Energy, kcal	5.5
Carbohydrate, g	0.21
Protein, g	0.28
Lipid, g	0.39
Sodium chloride, mg	0.20
Bisacurone, μg	400
Turmeronol A, μg	100
Turmeronol B, μg	100

CLE, *Curcuma longa* extracts.

### 2.6 Primary outcome domain

The primary outcome domain was cognitive function, which was assessed using the digital cognitive assessment tool Nou-Know (Eisai Co., Ltd., Tokyo, Japan), the Japanese version of the Montreal Cognitive Assessment (MoCA-J) (15), and the Trail Making Test (TMT) Parts A and B (16).

Nou-Know (Eisai Co., Ltd., Tokyo, Japan) is a tablet-based, self-administered cognitive assessment tool derived from the Cogstate Brief Battery (Cogstate Ltd., Melbourne, Australia) (17, 18), which has been validated in previous studies (19). It consists of four playing card–based tasks: Detection Task (psychomotor function), Identification Task (attention), One Card Learning Task (visual learning), and One Back Task (working memory). A Brain Performance Index is calculated relative to age-matched normative data, providing an estimate of brain age. Two composite scores are also generated: Composite A (concentration score; derived from the Detection Task and Identification Task), which reflects psychomotor function and attention through processing speed, and Composite B (memory score; derived from the One Card Learning Task and One Back Task), which reflects visual learning and working memory through both speed and accuracy. At each administration, card sequences in tasks are randomized to minimize practice effects. The same version of Nou-Know, including the same test battery and brain-age calculation algorithm, was used at both assessment time points. The brain age is used as a supportive, exploratory measure of age-adjusted cognitive performance rather than as a validated indicator of brain aging in MCI because the normative reference dataset used by Nou-Know was not specifically developed or validated for the MCI population.

### 2.7 Secondary endpoints

Changes in high-sensitivity C-reactive protein (hsCRP), tumor necrosis factor-α (TNF-α), and brain-derived neurotrophic factor (BDNF) were evaluated. hsCRP was measured by standard latex agglutination turbidimetry using N-Assay LA CRP-U Nittobo (Nittobo Medical Co., Ltd., Japan), TNF-α was measured using the Human TNF-alpha QuantiGlo enzyme-linked immunosorbent assay (ELISA) Kit (R&D Systems, Rochester, MN, USA), and BDNF was measured using the Human Free BDNF Quantikine ELISA Kit (R&D Systems, Rochester, MN, USA).

### 2.8 Study procedures

Participants were followed for 4 months and instructed to take two CLE capsules per day. Capsules could be taken at a time chosen by the participant to avoid disruption of usual routines and were to be swallowed without chewing.

Cognitive assessments were conducted at baseline and after 4 months at the outpatient department of the study site. Participants self-administered the Nou-Know tasks with assistance from medical assistants. The MoCA-J and TMT were administered by a certified public psychologist.

Safety monitoring included interviews, physical examinations, blood biochemistry tests, urinalysis, and evaluation of blood parameters at baseline, 2 months, and 4 months. Participant diaries were reviewed at each visit to assess adherence and identify any lifestyle changes. Table 2 summarizes the schedule of assessments.

**Table 2:** Schedule of assessments.

Assessment	Timepoint (months)		
	0	2	4
Clinical interview	●	●	●
Physical examination	●	●	●
Participant diary review	●	●	●
Blood parameters measurements	●	●	●
Cognitive function assessments			
Nou-Know	●		●
MoCA-J	●		●
TMT-A and B	●		●
Safety assessments			
Blood biochemistry tests/clinical chemistry	●	●	●
Urinalysis	●	●	●

MoCA-J, Japanese version of the Montreal Cognitive Assessment; TMT, Trail Making Test.

During the study period, participants were instructed not to initiate new medications that could affect cognitive function. If initiation of such treatment became necessary, the participant was withdrawn from the study. Participants already receiving treatment that could affect cognitive function at enrollment were allowed to stay on treatment but were prohibited from changing the dose; any dose modification resulted in withdrawal from the study. Initiation of new cognitive rehabilitation or physiotherapy was also prohibited. Participants with major protocol deviations were excluded from the Per Protocol Set (PPS).

### 2.9 Statistical analysis

The statistical analysis was performed in the PPS population, which included participants who completed the study in accordance with the predefined protocol and had no major protocol deviations. Results are presented as means (± standard deviation [SD]), 95% confidence intervals (CIs), and Cohen’s dz values as effect sizes (20). All analyses were performed using IBM SPSS Statistics (version 26; IBM, Armonk, NY, USA). Paired *t*-tests were used to compare outcomes at baseline and after CLE intake. No adjustment for multiple comparisons was performed, and the individual cognitive analyses were considered exploratory.

## 3 Results

### 3.1 Participants

A total of 13 individuals were enrolled. One participant declined to continue for personal reasons. In addition, two participants were excluded from the PPS analysis set because the intake rate of the CLE capsules was less than 60% (*n* = 1) and the change in lifestyle during the scheduled study period (*n* = 1). The analysis set consisted of 2 men and 8 women with a mean (±SD) age of 74.8 (± 6.1) years. The mean (± SD) intake rate of the CLE capsules was 93.2% (± 11.5%). The baseline characteristics of participants are shown in Table 3.

**Table 3:** Baseline characteristics of the participants.

Characteristic	*N* = 10	
	Mean	SD
Sex, *n*	Male: 2	
	Female: 8	
Age, years	74.8	6.1
BMI, kg/m^2^	20.7	3.8
SBP, mmHg	132.9	15.2
DBP, mmHg	75.2	15.1
Education, years	12.1	1.7
Living alone, *n*	0/10	

BMI, body mass index; DBP, diastolic blood pressure; SBP, systolic blood pressure; SD, standard deviations.

### 3.2 Nou-know scores

Nou-Know scores at baseline and 4 months are shown in Table 4. At month 4 of CLE intake, estimated brain age was significantly lower and memory scores (Composite B) were significantly higher compared with baseline (both *p* < 0.05) (Figures 1A,B). In contrast, focus scores (Composite A) did not differ significantly from baseline (*p* = 0.521).

**Table 4:** Nou-Know scores.

Outcome	Absolute values				Change from baseline			*p*-value	Cohen’s dz value
	Month 0		Month 4		Month 4				
	Mean	SD	Mean	SD	Mean	SD	95% CI		
Nou-Know									
Brain age	79.5	5.7	76.3*	6.7	−3.2	4.4	−6.35 to −0.01	0.049	−0.718
Focus score (Composite A)	87.9	11.9	86.4	11.0	−1.5	7.1	−6.62 to 3.60	0.521	−0.211
Memory score (Composite B)	90.0	8.5	97.4*	9.8	7.4	10.2	0.104 to 14.72	0.047	0.726

CLE, *Curcuma longa* extracts; CI, confidence intervals; SD, standard deviations.

Values represent the means and standard deviations at 0 and 4 months for 10 participants. Differences from baseline were evaluated by paired *t*-tests (^*^p < 0.05).

**Figure 1:**
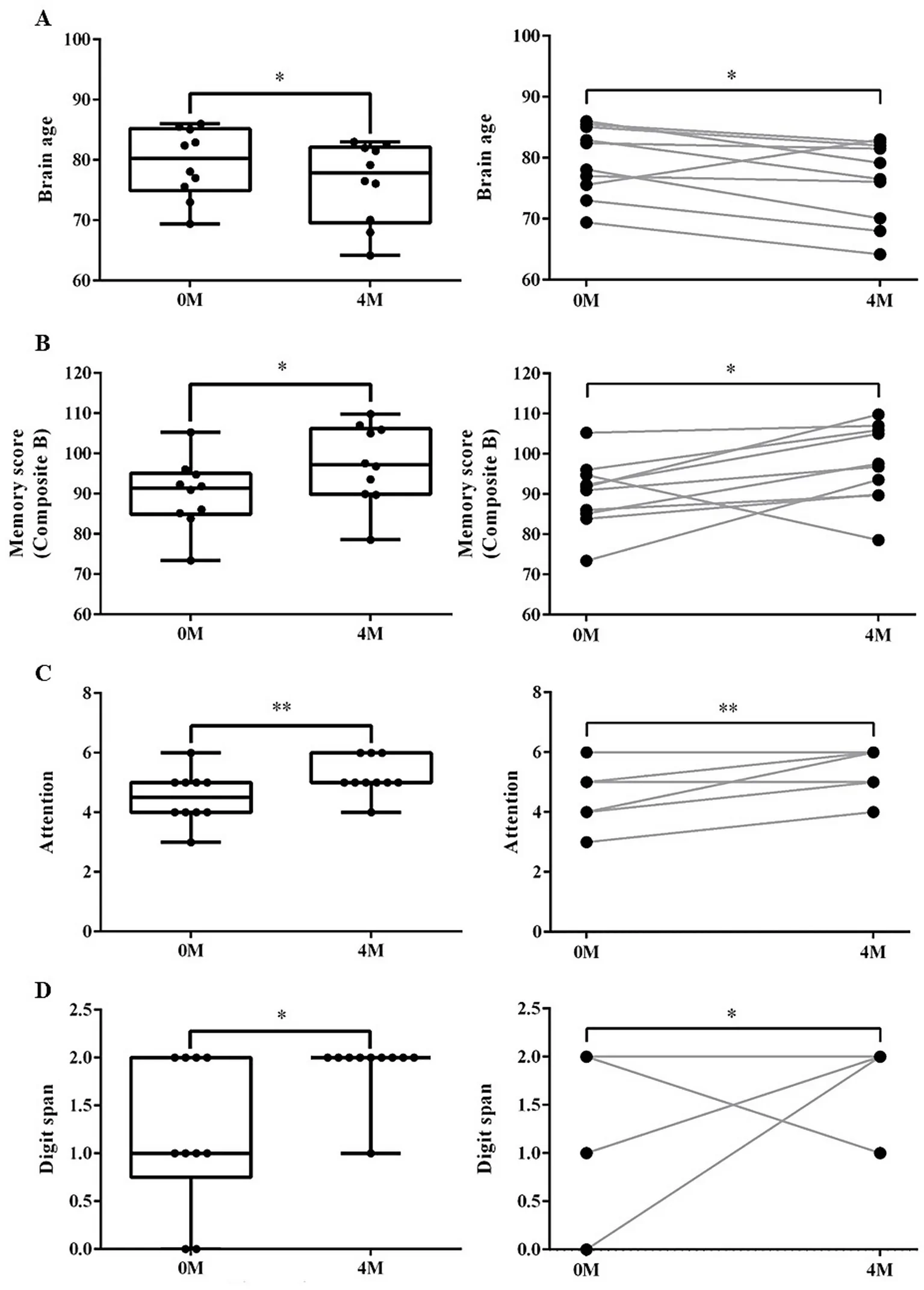
Box-and-whisker plots and spaghetti plots of cognitive function measures from baseline (0 M) to month 4 (4 M) during the observation period. **(A)** Nou-Know brain age, **(B)** Nou-Know memory score (Composite B), **(C)** MoCA-J attention, and **(D)** MoCA-J digit span. In the box-and-whisker plots, boxes denote the interquartile range, center lines denote the median, whiskers denote the full data range, and dots represent individual participants (*n* = 10). In the spaghetti plots, dots indicate individual participants and gray lines connect repeated measurements from the same participant (*n* = 10). Differences from baseline were evaluated by paired *t*-tests (*p < 0.05, **p < 0.01). CLE, *Curcuma longa* extracts; MoCA-J, Japanese version of the Montreal Cognitive Assessment.

### 3.3 MoCA-J scores

At month 4 of CLE intake, mean MoCA-J total scores did not differ significantly from baseline (18.8 ± 3.1 vs. 19.0 ± 3.1, *p* = 0.716) (Table 5). Subscale analysis showed that both the attention domain score (*p* < 0.01) and the digit span test score (*p* < 0.05) were significantly higher compared with baseline (Figures 1C,D), whereas no significant differences were observed in any other subscales.

**Table 5:** MoCA-J scores.

Outcome	Absolute values				Change from baseline			*p*-value	Cohen’s dz value
	Month 0		Month 4		Month 4				
	Mean	SD	Mean	SD	Mean	SD	95% CI		
MoCA-J									
Total score	18.8	3.1	19	3.1	0.2	1.7	−1.01 to 1.41	0.716	0.119
Subcategory									
Visuospatial abilities/executive function	3.7	0.7	3.3	0.9	−0.4	0.7	−0.90 to 0.10	0.104	−0.572
Alternating trail making	0.5	0.5	0.4	0.5	−0.1	0.3	−0.33 to 0.13	0.343	−0.316
Visuoconstruction skills (cube)	0.7	0.5	0.6	0.5	−0.1	0.6	−0.51 to 0.31	0.591	−0.176
Visuoconstruction skills (clock)	2.5	0.5	2.3	0.8	−0.2	0.8	−0.76 to 0.36	0.443	−0.254
Naming	3.0	0.0	3.0	0.0	0.0	0.0	NE	NE	NE
Attention	4.5	0.8	5.2**	0.6	0.7	0.7	0.22 to 1.18	0.010	1.037
Digit span	1.2	0.8	1.9*	0.3	0.7	0.9	0.02 to 1.38	0.045	0.738
Vigilance	1.0	0.0	0.9	0.3	−0.1	0.3	−0.33 to 0.13	0.343	−0.316
Serial sevens	2.3	0.8	2.4	0.7	0.1	0.9	−0.53 to 0.73	0.726	0.114
Language	4.0	0.5	4.1	0.6	0.1	0.3	−0.13 to 0.33	0.343	0.316
Sentence repetition	1.0	0.5	1.1	0.6	0.1	0.3	−0.13 to 0.33	0.343	0.316
Verbal fluency	0.2	0.4	0.3	0.5	0.1	0.6	−0.31 to 0.51	0.591	0.176
Abstraction	1.3	0.7	1.7	0.5	0.4	0.7	−0.10 to 0.90	0.104	0.572
Delayed recall	0.1	0.3	0.1	0.3	0.0	0.0	NE	NE	NE
Orientation	4.1	2.1	3.4	1.0	−0.7	1.5	−1.77 to 0.37	0.173	−0.468

CLE, *Curcuma longa* extracts; CI, confidence intervals; MoCA-J, Japanese version of the Montreal Cognitive Assessment; NE, not estimable; SD, standard deviations.

Values represent the means and standard deviations at 0 and 4 months for 10 participants. Differences from baseline were evaluated by paired *t*-tests (*p < 0.05, **p < 0.01). NE indicates that *p*-value could not be calculated because all observations had the same value at 0 and 4 months.

### 3.4 TMT completion times

Completion times for the TMT after CLE intake did not differ significantly from baseline for either Part A (*p* = 0.665) or Part B (*p* = 0.592) (Table 6).

**Table 6:** TMT completion times.

Outcome	Absolute values				Change from baseline			*p*-value	Cohen’s dz value
	Month 0		Month 4		Month 4				
	Mean	SD	Mean	SD	Mean	SD	95% CI		
TMT part A, sec	56.8	16.6	59.3	22.3	2.50	16.7	−10.3 to 15.3	0.665	0.142
TMT part B, sec	166.9	81.8	150.1	71.1	−16.8	90.5	−86.4 to 52.7	0.592	−0.176

CLE, *Curcuma longa* extracts; CI, confidence intervals; SD, standard deviations; TMT, Trail Making Test.

Values represent the means and standard deviations at 0 and 4 months for 10 participants. Differences from baseline were evaluated by paired *t*-tests.

### 3.5 Blood parameters

No significant differences from baseline were observed in hsCRP, TNF-α, or BDNF (data not shown).

### 3.6 Safety

No significant differences from baseline were observed in safety parameters, including hematology, blood biochemistry, urinalysis, or physical examination findings. Individual safety data were reviewed by the study physician, and no medically relevant changes associated with the test food were identified.

## 4 Discussion

We conducted a 4-month, single-arm study to observe the effects of daily intake of a mixture of hot water extract and supercritical carbon dioxide extract of *Curcuma longa* (CLE) containing turmeronol A and B and bisacurone on cognitive function and related blood parameters in older adults with MCI due to AD while awaiting AAT. CLE intake was associated with significantly higher memory scores (Composite B) and significantly lower estimated brain age at 4 months compared with baseline, as measured by the Nou-Know (Figures 1A,B). MoCA-J total scores did not differ significantly after CLE intake, but subscale scores for the attention domain and digit span test increased significantly from baseline (Figures 1C,D). No significant differences from baseline were observed in hsCRP, TNF-α, or BDNF levels.

Among the cognitive measures used, the Nou-Know Composite B and MoCA-J digit span test scores primarily reflect working memory function (21, 22). Working memory is a core cognitive function of the frontal lobe that involves the temporary storage and manipulation of information (23). The observed significant changes in these measures by CLE may be associated with changes in cognitive processes involving the frontal lobe, particularly working memory, in individuals with MCI due to AD.

In contrast, no significant changes were observed in TMT Parts A or B, which also reflect working memory function (Table 6). This discrepancy may be explained by differences in cognitive demands across tasks. The digit span test primarily assesses short-term and working memory, whereas the TMT requires more complex, high-load cognitive processing, including selective and divided attention, cognitive flexibility, visual search, processing speed, and visual-motor tracking (16). These differences may explain the lack of significant changes in TMT completion times, in contrast to the changes in digit span test scores, which reflect a more limited aspect of attention. Although TMT and the MoCA-J assess different aspects of cognitive function, these results also suggest that the effect of CLE on overall cognitive function may be limited. Therefore, the improvement in the attention-related subscore should be interpreted cautiously and may not necessarily reflect an overall improvement in cognitive function.

The brain age score used in the Nou-Know test was developed based on validated computerized cognitive assessments and age-stratified normative data to express an age-adjusted measure of cognitive performance and has been shown to distinguish cognitively normal individuals from those with memory impairment and showed good test–retest reliability (24, 25). However, the clinical significance of a specific magnitude of change in brain age has not yet been established. Therefore, in the present study, the 3.2-year reduction in brain age observed after CLE intake should not be interpreted as an actual rejuvenation of brain age, but rather as a change in age-adjusted cognitive performance.

Previous studies have shown that poorer performance on measures of working memory and attention, including digit span, is associated with greater difficulties in instrumental activities of daily living (IADL; e.g., preparing meals, taking medications, managing money, shopping for groceries, performing light housework, using a telephone, and driving during the day or at night), as well as more depressive symptoms and sleep complaints in older adults (26, 27). However, in the present study, the extent to which a 0.7-point increase in digit span following CLE intake is associated with meaningful improvements in these outcomes remains unclear. Further clinical studies incorporating measures of daily functioning and related clinical outcomes are warranted to clarify the clinical relevance of this magnitude of change.

Practice effects may also influence the results of cognitive tests, where repeated administration of tests over short intervals can lead to improved performance (28). Among MoCA subtasks in the attention domain, the digit span test may be particularly susceptible to practice effects (29). To mitigate this, assessments were limited to two time points (baseline and 4 months). The Nou-Know assessment is designed to minimize practice effects through randomized task presentation. Although practice effects cannot be completely excluded, the observed significant changes from baseline in the Nou-Know estimated brain age and memory scores (Composite B) suggest that CLE intake may be associated with changes in working memory performance in individuals with MCI due to AD.

Notably, significant changes from baseline were observed in the memory score (Composite B), which reflects visual learning and working memory, but not in the focus score (Composite A), which reflects psychomotor function and attention, in the Nou-Know test. These findings suggest that CLE intake may be associated with changes in the accuracy, rather than the speed, of cognitive task performance. However, these phenotypic changes were not accompanied by significant changes in blood parameters. In the absence of supportive biochemical evidence or biomarker data, the potential mechanisms by which CLE intake may influence cognitive function remain unclear.

Chronic inflammation contributes to the development of MCI and AD, partly by affecting the expression of BDNF, which plays an important role in neuronal survival and synaptic plasticity (30, 31). Preclinical animal studies have shown that water extract of *C. longa*, including turmeronols A and B, can suppress the production of inflammatory mediators and microglial activation through inhibition of NF-kB signaling, attenuate pathological alteration in the brain, and improve cognitive functions in Aβ_1-42_-induced AD model mice (8). In addition, previous clinical studies suggested CLE may have beneficial effects on cognitive function, including attention (11). These previous reports raise the possibility that CLE may influence neuroinflammatory processes. However, in the present study, no significant changes were observed in the circulating levels of hsCRP, TNF-α, or BDNF. Accordingly, the present findings do not provide evidence that the observed cognitive changes were mediated by anti-inflammatory or BDNF-related mechanisms. A previous clinical study reported no significant associations between brain neuroinflammation detected by translocator protein-positron emission tomography (TSPO-PET) and blood inflammatory cytokine levels in individuals with MCI and AD (32). Thus, the absence of changes in circulating hsCRP and TNF-α in the present study does not necessarily exclude a potential influence of CLE on neuroinflammatory processes in the brain. Further studies will be needed to clarify the influence of CLE on neuroinflammation using TSPO-PET in clinical studies.

This study has several limitations. First, the single-arm design, absence of a placebo or control group, small sample size, and short follow-up period limit the strength of the findings and restrict causal interpretation. In particular, it is difficult to clearly determine whether the observed changes in cognitive performance were specifically associated with CLE intake or were attributable to other factors, including residual practice effects resulting from repeated administration of cognitive tests. Second, three of the 13 enrolled participants were excluded from the final analysis, leaving a final sample size of 10 participants. In addition, 21 cognitive endpoints were assessed. Therefore, the statistical power was limited, the exclusion of participants may have introduced selection bias, the generalizability of the findings to the broader population of individuals with MCI due to AD may be limited, and the findings should be considered exploratory. Third, CLE was administered at a fixed dose, precluding evaluation of dose–response relationships. Despite these limitations, the present study provides preliminary evidence of a potential association between CLE intake and changes in working memory in individuals with MCI due to AD. Further investigation in larger, placebo-controlled studies is warranted to evaluate the effects of different doses and durations of CLE intake.

## Data Availability

The raw data supporting the conclusions of this article will be made available by the authors, without undue reservation.
